# Social and demographic predictors of no transport prior to premature cardiac death: United States 1999–2000

**DOI:** 10.1186/1471-2261-6-45

**Published:** 2006-11-15

**Authors:** Elizabeth Barnett, Steven Reader, Beverly G Ward, Michele L Casper

**Affiliations:** 1 Department of Epidemiology and Biostatistics, University of South Florida, Tampa, USA; 2 Department of Geography, University of South Florida, Tampa, USA; 3 Department of Anthropology, University of South Florida, Tampa, USA; 4 Centers for Disease Control and Prevention, Atlanta, USA

## Abstract

**Background:**

In the United States, over one-third of premature cardiac deaths occur outside of a hospital, without any transport prior to death. Transport prior to death is a strong, valid indicator of help-seeking behavior. We used national vital statistics data to examine social and demographic predictors of risk of no transport prior to cardiac death. We hypothesized that persons of lower social class, immigrants, non-metropolitan residents, racial/ethnic minorities, men, and younger decedents would be more likely to die prior to transport.

**Methods:**

Our study population consisted of adult residents of the United States, aged 25 to 64 years, who died from heart disease during 1999–2000 (n = 242,406). We obtained transport status from the *place of death *variable on the death certificate. The independent effects of social and demographic predictor variables on the risk of a cardiac victim dying prior to transport vs. the risk of dying during or after transport to hospital were modeled using logistic regression.

**Results:**

Results contradicted most of our *a priori *hypotheses. Persons of lower social class, immigrants, most non-metropolitan residents, and racial/ethnic minorities were all at *lower risk *of dying prior to transport. The greatest protective effect was found for racial/ethnic minority decedents compared with whites. The strongest adverse effect was found for marital status: the risk of dying with no transport was more than twice as high for those who were single (OR 2.35; 95% CI 2.29–2.40) or divorced (OR 2.29; 95% CI 2.24–2.34), compared with married decedents. Geographically, residents of the Western United States were at a 47% increased risk of dying prior to transport compared with residents of the metropolitan South.

**Conclusion:**

Our results suggest that marital status, a broad marker of household structure, social networks, and social support, is more important than social class or race/ethnicity as a predictor of access to emergency medical services for persons who suffer an acute cardiac event. Future research should focus on ascertaining "event histories" for all acute cardiac events that occur in a community, with the goal of identifying the residents most susceptible to cardiac fatalities prior to medical intervention and transport.

## Background

Secondary prevention efforts for heart disease mortality focus on improving access to medical care, including emergency medical services, and reducing case fatality rates for persons who suffer a major cardiac event [1,2]. When major cardiac events strike men and women in the prime of life (young adulthood and middle age), secondary prevention efforts are particularly critical to minimize the adverse effects for patients, their families and dependents, and society as a whole.

In the United States, over one-third of premature (i.e. prior to age 65 years) cardiac deaths occur outside of a hospital, without any transport prior to death [3]. Transport prior to death is a strong and valid indicator of help-seeking behavior, whether by the cardiac victim or a family member or bystander. While some unknown fraction of "no transport" cardiac decedents may have received medical intervention, the assumption is that most did not. Moreover, these "no transport" cardiac deaths may be heterogeneous in etiology (e.g. sudden cardiac arrest, myocardial infarction, or congestive heart failure) and consequently in the degree to which medical intervention might have postponed fatality. One body of research has made the assumption that any cardiac death occurring "out of hospital" was in fact the result of a sudden cardiac arrest (SCA), in which death typically follows the onset of symptoms very rapidly (< 1 hour), and for which medical intervention was assumed to be of limited efficacy [4,5]. More recently, efforts to prevent SCA deaths have included the development and widespread deployment of automatic external defibrillators (AEDs); and public education campaigns by the American Heart Association, the Centers for Disease Control and Prevention, and the National Heart, Lung, and Blood Institute have focused attention on the life-saving potential of rapid access to cardiopulmonary resuscitation (CPR) and AEDs [6]. While there has been a recent focus on increasing lay witness intervention in cases of cardiac arrest [7], most bystanders are unprepared or unwilling to intervene [8]. Consequently, rapid arrival of trained emergency medical services personnel offers the best hope of survival to SCA victims [9,10].

"No transport" cardiac deaths may also result from myocardial infarctions (MIs) in which time from onset of symptoms to death may be quite lengthy, but delays in seeking medical intervention and barriers to obtaining treatment result in case fatality [11,12]. These barriers include patient knowledge, socioeconomic status, geographic location, lack of transportation, and delays in emergency medical services (EMS) response times [12-15].

Secondary prevention of cardiac fatalities requires reducing the proportion of cardiac events that receive delayed or no medical intervention [2,9,16]. Obviously, cardiac deaths occurring in the hospital are no better an outcome for patients or society than deaths occurring prior to transport. In addition, medical intervention may be of little use in some cases – for example cardiac arrest resulting from non-shockable rhythms (e.g. asystole) [17]. However, the fundamental assumption underlying this research is that reducing deaths prior to transport will reduce the overall case fatality rate: transport and medical intervention should help at least some patients survive to hospital discharge [18].

In this study, we used vital statistics data to examine social and demographic predictors of risk of no transport prior to cardiac death for the years 1999–2000, for the entire United States. Only one previous study similar to ours has been published, and that study used sample data for a far smaller number of decedents, for the period 1979–1989 [19]. Given well-known disparities in access to medical care for disadvantaged sub-populations in the U.S., we hypothesized that persons of lower social class, immigrants, non-metropolitan residents, and racial/ethnic minorities would all be more likely to die prior to transport. In addition, we hypothesized greater likelihood of death prior to transport for men vs. women (because men have been shown to delay seeking treatment), and younger decedents vs. older decedents (because acute heart disease symptoms are more likely to be missed, misinterpreted, and misdiagnosed in younger adults).

## Methods

### Study population and definitions

Our study population consisted of all adult residents of the United States, aged 25 to 64 years old, who died from heart disease during the years 1999–2000. We examined death certificate data for 242,406 decedents. Death certificate data files were obtained from the National Center for Health Statistics. A cardiac death was defined as any death for which the underlying cause coded on the death certificate was *diseases of the heart *(ICD 10 codes I00-I09, I11, I13, I20-I51) or *symptoms, signs, and ill-defined conditions *(ICD 10 codes R96-R99). We used a broad inclusive definition of heart disease because previous research has shown misclassification in cause-of-death coding for heart disease [20].

We included the cause of death category *symptoms, signs, and ill-defined conditions *(SSID) for several reasons. These codes are used by physicians, coroners, and medical examiners when there is insufficient post-mortem evidence to support assigning a specific disease as cause of death [21]. It should be noted that deaths resulting from any kind of injury or external cause are not coded to SSID. Earlier research on sudden cardiac arrest fatalities indicated that these deaths were sometimes coded to SSID on the death certificate [4]. Based on the presumption that the vast majority of sudden cardiac arrest deaths resulted from underlying heart disease (as opposed to injury or trauma), previous studies found that heart disease death rates for which SSID deaths had been excluded from the numerator were significantly underestimated in some populations [4]. A study of MONICA (Multinational Monitoring of Trends and Determinants in Cardiovascular Disease) data from Belgium found that approximately 5% of "definite" or "possible" cases of acute myocardial infarction had been coded to SSID on the death certificate [20].

Under ICD-10, which was implemented in 1999, cardiac arrest is considered to be an ill-defined condition [22,23]. In the case of a cardiac arrest, if any other specific condition is listed on the death certificate the underlying cause will be coded as that specific condition and not cardiac arrest. Therefore, deaths for which cardiac arrest *was *coded as the underlying cause (ICD 10 code I46), were deaths for which no other disease information was available.

Our outcome in this study was no transport prior to cardiac death. We obtained transport status information for each decedent from the *place of death *variable on the death certificate. We categorized a cardiac death as occurring with no transport if place of death was reported as one of the following: (1) at home; (2) in a nursing home; (3) at another location in the community. Deaths that occurred during or after transport had place of death reported as one of the following: (1) dead on arrival [at hospital]; (2) emergency room/outpatient; (3) hospital: in-patient; (4) hospital: status unknown [inpatient vs. outpatient status]. It should be noted that fewer than 1% of the cardiac deaths in our study had "dead on arrival" listed as the place of death.

Social and demographic characteristics of decedents, examined as predictors of no transport prior to cardiac death, were also obtained from the death certificate. We examined age at time of death, gender, race and ethnicity (Hispanics, white non-Hispanics, black non-Hispanics, Asian non-Hispanics and American Indian/Alaska Native non-Hispanics), social class (measured by educational attainment), immigrant status (derived from country of birth variable), marital status, region of residence (Northeast, Midwest, South, or West), and rurality of residence (measured by metropolitan vs. non-metropolitan county of residence).

### Analytic methods

We first examined the distribution of each sociodemographic predictor variable for all cardiac decedents, for decedents who died prior to transport, and for decedents who died after transport. Then, we used logistic regression analysis to model the independent effects of each of the social and demographic predictor variables on the risk of a cardiac victim dying prior to transport to hospital (''no transport deaths'') vs. the risk of dying during or after transport to hospital. Specifically, we examined 8 categories of decedent age, men vs. women, 5 racial/ethnic groups, 4 marital status groups, high school diploma vs. no diploma, foreign born vs. U.S born, and 8 geographic areas defined by region and rurality. Odds ratios and 95% confidence intervals for each unique value of each predictor variable were obtained from the logistic regression model.

Subsequently, we conducted a second logistic regression analysis to examine joint effects of three of our important predictor variables: marital status, gender, and race/ethnicity. A dummy variable was created which had as its values every unique combination of marital status, gender, and race/ethnicity (i.e. 40 unique values). This approach provided separate odds ratios and 95% confidence intervals for each of these 40 demographic groups of cardiac decedents. The model was also adjusted for age, social class, immigrant status, region and rurality.

### Human subjects

This study was approved by the Social and Behavioral Science Institutional Review Board of the University of South Florida.

## Results

During 1999–2000, approximately 1.5 million heart disease deaths occurred in the United States. Of these decedents, almost a quarter of a million died prematurely (i.e. before 65 years of age). Our study population included 242,406 cardiac decedents who were residents of the United States, died in the U.S., and were 25 to 64 years old at the time of their deaths. Death prior to transport occurred for 37% of these decedents.

Table 1 provides detailed social and demographic characteristics of our study population. The age distribution of these premature cardiac decedents was skewed, as expected, with only 3.3% of decedents aged 25–34 years old vs. 53.4% aged 55 to 64 years old at the time of death. The majority of decedents were male (69.5%), white non-Hispanic (72.3%), high school graduates (91.1%) and married at the time of their deaths (52.5%). Relatively few of the decedents were Asian (n = 3,888) or American Indian/Alaska Native (n = 1,595); however we included all racial/ethnic groups in all of our analyses. Persons born abroad comprised 7.4% of all decedents. Geographically, the decedents were concentrated, as expected, in metropolitan counties (76.3%). Regionally, the greatest proportion of decedents resided in the South (41%), with the lowest proportion in the West (17.3%).

**Table 1:** Characteristics of premature cardiac decedents (25–64 years old), United States, 1999–2000

	**Death Prior to Transport** ** n = 89,408**	**Death After Transport** ** n = 152,998**	**Total** ** n = 242,406**
**Age at Death:**			
25–34 years	3,100 (3.5%)	5,006 (3.3%)	8,106 (3.3%)
35–44 years	11,915 (13.3%)	18,836 (12.3%)	30,751 (12.7%)
45–54 years	27,852 (31.2%)	46,283 (30.3%)	74,135 (30.6%)
55–64 years	46,541 (52.1%)	82,873 (54.2%)	129,414 (53.4%)
			
**Gender:**			
Male	63,693 (71.2%)	104,881 (68.6%)	168,574 (69.5%)
Female	25,715 (28.8%)	48,117 (31.5%)	73,832 (30.5%)
			
**Race and Hispanic Ethnicity:**			
White, non-Hispanic	67,688 (75.7%)	107,621 (70.3%)	175,309 (72.3%)
Black, non-Hispanic	15,859 (17.7%)	32,712 (21.4%)	48,571 (20.0%)
Hispanic	4,131 (4.6%)	8,912 (5.8%)	13,043 (5.4%)
Asian, non-Hispanic	1,092 (1.2%)	2,796 (1.8%)	3,888 (1.6%)
AI/AN, non-Hispanic	638 (0.7%)	957 (0.6%)	1,595 (0.7%)
			
**Social Class:**			
Not high school graduate	7,612 (8.5%)	14,067 (9.2%)	21,679 (8.9%)
High school graduate	81,796 (91.5%)	138,931 (90.8%)	220,727 (91.1%)
			
**Immigrant Status:**			
Foreign-born	5,636 (6.3%)	12,347 (8.1%)	17,983 (7.4%)
Not foreign-born	83,772 (93.7%)	140,651 (91.9%)	224,423 (92.6%)
			
**Marital Status at Time of Death:**			
Married	36,185 (40.5%)	91,176 (59.6%)	127,361 (52.5%)
Widowed	6,799 (7.6%)	10,170 (6.7%)	16,969 (7.0%)
Divorced	27,024 (30.2%)	29,467 (19.3%)	56,491 (23.3%)
Single	19,400 (21.7%)	22,185 (14.5%)	41,585 (17.2%)
			
**Residence at Time of Death:**			
Metro South	24,012 (26.9%)	44,570 (29.1%)	68,582 (28.3%)
Metro Northeast	14,606 (16.3%)	25,585 (16.7%)	40,191 (16.6%)
Metro Midwest	14,295 (16.0%)	26,532 (17.3%)	40,827 (16.8%)
Metro West	15,788 (17.7%)	19,567 (12.8%)	35,355 (14.6%)
Non-Metro South	10,602 (11.9%)	20,513 (13.4%)	31,115 (12.8%)
Non-Metro Northeast	1,613 (1.8%)	3,248 (2.1%)	4,861 (2.0%)
Non-Metro Midwest	5,557 (6.2%)	9,412 (6.2%)	14,969 (6.2%)
Non-Metro West	2,935 (3.3%)	3,571 (2.3%)	6,506 (2.7%)
			
**Underlying Cause on Death Certificate:**			
Ischemic heart disease	58,389 (65.3%)	103,148 (67.4%)	161,537 (66.6%)
Other forms of heart disease	11,868 (13.3%)	29,138 (19.1%)	41,006 (16.9%)
Hypertensive heart disease	6,138 (6.9%)	6,310 (4.1%)	12,448 (5.1%)
Heart failure	2,609 (2.9%)	3,925 (2.6%)	6,534 (2.7%)
Ill-Defined Conditions (inc. cardiac arrest)	10,404 (11.6%)	10,477 (6.9%)	20,881 (8.6%)

To avoid misclassification bias in our study, we chose a broad inclusive definition of heart disease (diseases of the heart) and also included SSID deaths as cardiac deaths. Therefore, we examined the distribution of several specific causes of heart disease death within our study population (Table 1). For these statistics, we included cardiac arrest (ICD-10: I46) as an ill-defined condition, per the coding philosophy of ICD-10 [22]. The majority of decedents had ischemic heart disease (ICD-10: I20-I25) listed as the underlying cause of death (66.6%). Hypertensive heart disease (ICD-10: I11) accounted for 5.1% of the deaths and heart failure (ICD-10: I50) accounted for 2.7% of the deaths. The category "other forms of heart disease", which comprised 16.9% of these deaths, included rheumatic heart disease (ICD-10: I00-I09), endocarditis (ICD-10: I33), diseases of the pericardium and myocarditis (ICD-10: I30-31, I41), and several other less common forms of heart disease (ICD-10: I26-I28, I34-I38, I42-I45, I47-I49, I51). Symptoms, signs, and ill-defined conditions (ICD-10: R96-R99, I46) were listed as the underlying cause for 8.6% of the deaths.

**Figure 1:**
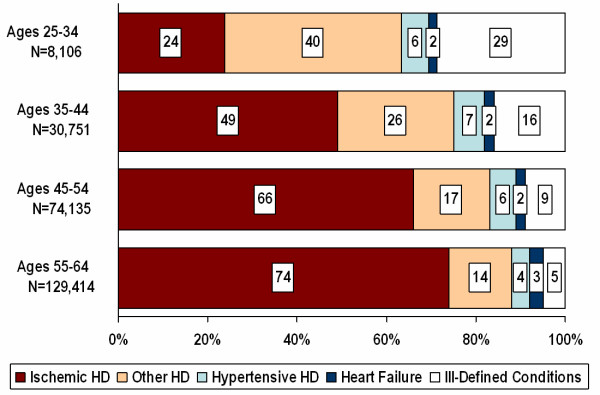
Specific causes of death for cardiac decedents, United States 1999–2000.

In addition, we examined specific causes of cardiac death by age within our study population (Figure 1). The greatest differences by age were observed for ischemic heart disease, other forms of heart disease, and SSID. Among the majority of decedents who were 55–64 years old at the time of their death, 74% died from ischemic heart disease, 14% from other forms of heart disease, and only 5% had their deaths coded to SSID. In contrast, the youngest decedents, who were 25–34 years old at death, were much more likely to have their death coded to an ill-defined condition (29%), and only 24% of these younger decedents had ischemic heart disease listed as the underlying cause.

Logistic regression analysis revealed several important independent sociodemographic predictors of risk of no transport prior to cardiac death (Table 2). However, results contradicted almost all of our *a priori *hypotheses. Persons of lower social class, immigrants, most non-metropolitan residents, and racial/ethnic minorities were all at *lower risk *of dying prior to transport compared with their respective referent groups. There was little effect of decedent age on risk of dying prior to transport. The greatest protective effect was found for most racial/ethnic minority decedents compared with whites; odds ratios for dying with no transport were 0.71 for Blacks (95% CI 0.70–0.73), 0.70 for Hispanics (95% CI 0.67–0.73) and 0.61 for Asians (95% CI 0.56–0.66).

**Table 2:** Odds ratios for risk of no transport vs. death after transport, for premature cardiac decedents, United States, 1999–2000

Sociodemographic Factor	Odds Ratio (95% CI)
**Age**	
25 – 29	0.94 (0.87 – 1.02)
30 – 34	0.92 (0.86 – 0.99)**
35 – 39	0.99 (0.95 – 1.03)
40 – 44	1.03 (0.99 – 1.07)
45 – 49	1.02 (1.00 – 1.05)
50 – 54	1.02 (0.99 – 1.05)
55 – 59	1.01 (0.98 – 1.03)
60 – 64 (referent)	1.00
	
**Gender**	
Male	1.16 (1.14 – 1.18)**
Female (referent)	1.00
	
**Race/ethnicity**	
Black	0.71 (0.70 – 0.73)**
Hispanic	0.70 (0.67 – 0.73)**
Asian	0.61 (0.56 – 0.66)**
American Indian	0.91 (0.82 – 1.01)
White (referent)	1.00
	
**Marital Status**	
Single	2.35 (2.29 – 2.40)**
Widowed	1.88 (1.81 – 1.94)**
Divorced	2.29 (2.24 – 2.34)**
Married (referent)	1.00
	
**Social class**	
Not a high school graduate	0.95 (0.92 – 0.98)**
High school graduate (referent)	1.00
	
**Immigrant Status**	
Foreign Born	0.91 (0.88 – 0.95)**
U.S. Born (referent)	1.00
	
**Region and Rurality**	
Non-metro Northeast	0.86 (0.81 – 0.91)**
Metro Northeast	1.02 (0.99 – 1.05)
Non-metro Midwest	1.03 (0.99 – 1.07)
Metro Midwest	0.94 (0.92 – 0.97)**
Non-metro West	1.47 (1.39 – 1.55)**
Metro West	1.47 (1.43 – 1.51)**
Non-metro South	0.96 (0.93 – 0.99)**
Metro South (referent)	1.00

The strongest predictor of risk of dying prior to transport was marital status. Compared with decedents who were married, the risk of dying with no transport was more than twice as high for those who were single (OR 2.35; 95% CI 2.29–2.40) or divorced (OR 2.29; 95% CI 2.24–2.34). Decedents who were widowed were also at increased risk of dying prior to transport (OR 1.88; 95% CI 1.81–1.94). Men were slightly more likely than women to die prior to transport (OR 1.16; 95% CI 1.14–1.18). Geographically, residents of both non-metropolitan and metropolitan counties in the West were at a 47% increased risk of dying prior to transport compared with residents of the metropolitan South (Table 2).

**Figure 2:**
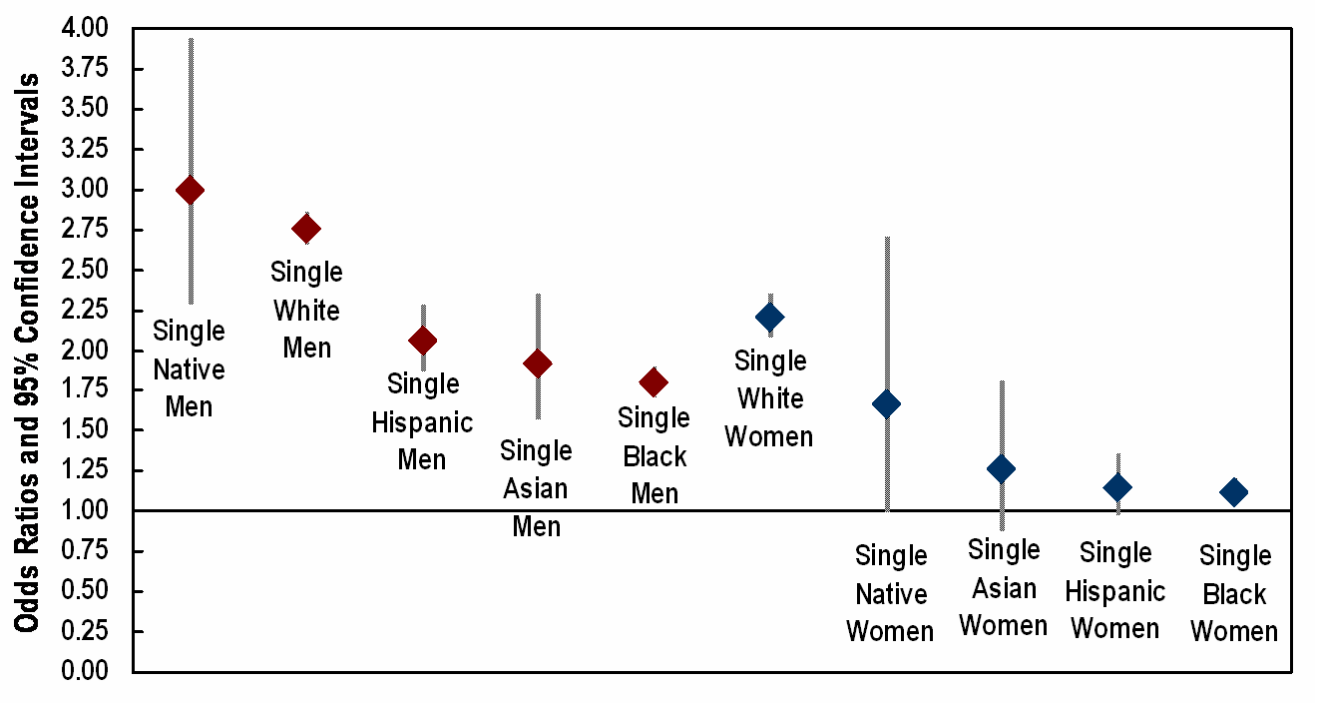
Relative risk of no transport prior to cardiac death: results for single men and women. Referent group = married white men. Logistic model adjusted for age, education, immigrant status, region, and metropolitan status of county of residence.

**Figure 3:**
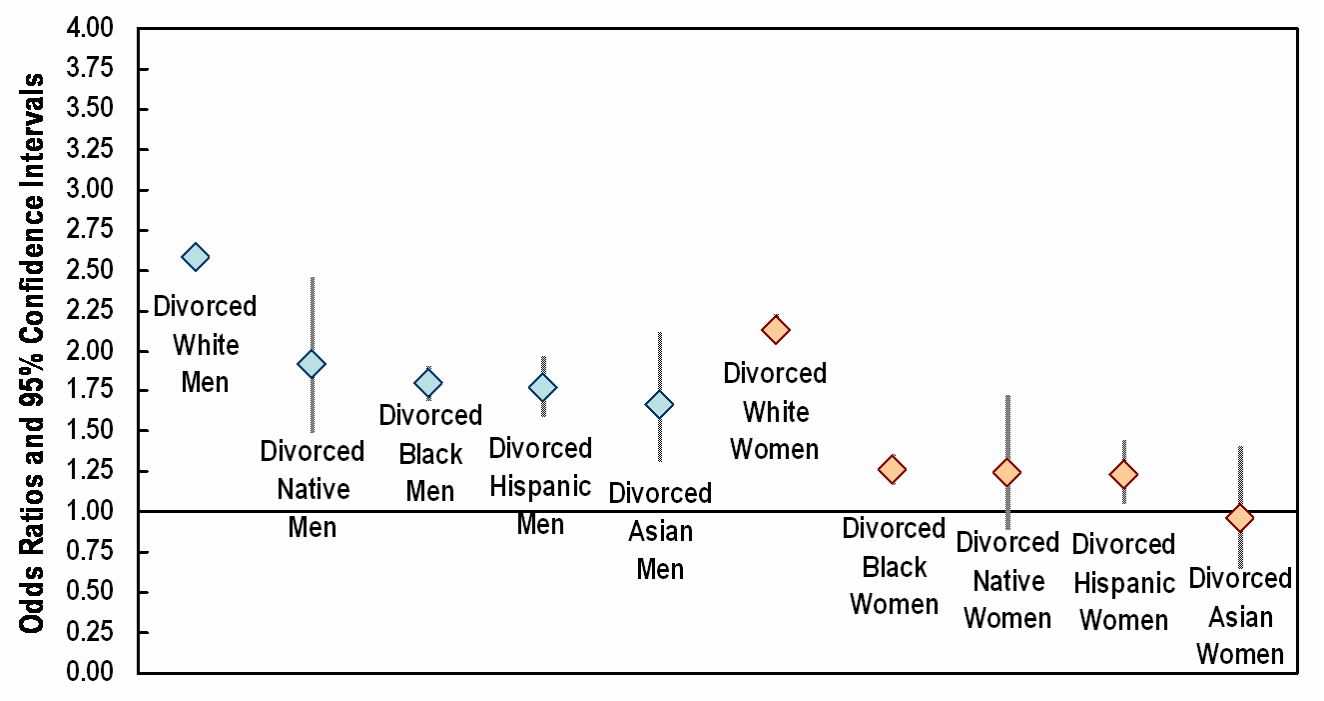
Relative risk of no transport prior to cardiac death: results for divorced men and women. Referent group = married white men. Logistic model adjusted for age, education, immigrant status, region, and metropolitan status of county of residence.

Relative risks of dying prior to transport for important demographic groups, defined by gender, race/ethnicity, and marital status, are shown in Figures 2 and 3. The referent group was married white men. Among persons who were single at the time of their death (Figure 2), the greatest risk of dying prior to transport was experienced by American Indian/Alaska Native men (OR = 3.0) and white men (OR = 2.8). Risks were somewhat lower but still elevated for Hispanic men (OR = 2.1), Asian men (OR = 1.9), and Black men (OR = 1.8). Among single women, the greatest excess risk was found for white women (OR = 2.2) and American Indian/Alaska Native women (OR = 1.7). There was only a slightly elevated risk of dying prior to transport for Asian, Hispanic, and Black women. Results for persons who were divorced at the time of their death were very similar to the results for single persons (Figure 3). Among divorced women, only white women had a markedly increased risk of dying prior to transport (OR = 2.1).

## Discussion

Taken as a whole, our results suggest that marital status, which is a broad marker of household structure, social networks, and social support, is more important than social class or race/ethnicity as a predictor of access to emergency medical services for persons who suffer an acute cardiac event. The majority of life-threatening cardiac events that occur in the community occur in a private home [16,18]. Persons who live alone and suffer a cardiac arrest will obviously not be capable of calling 911 or seeking aid on their own behalf, although agonal breathing and consciousness may continue for a few minutes following cardiac arrest [24]. Persons who live alone and suffer a myocardial infarction or other acute cardiac emergency may delay seeking medical aid or be physically or cognitively unable to seek aid. We can take marital status as an imperfect proxy of an individual's household living arrangements. Data from the census confirm that only a tiny minority of married persons live alone. In our study, persons who were unmarried were at greatest risk of dying prior to transport vs. dying in hospital. When we calculated odds ratios separately for population groups defined by gender, marital status, and race/ethnicity, we found variation in the magnitude of the excess risk. We interpret these variations to reflect unmeasured variation in household structure not captured by marital status. For example, odds ratios for single and divorced men of all racial/ethnic groups were higher than comparable odds ratios for women. This likely reflects the fact that unmarried women are more likely to live with children or other adult family members than their male counterparts [25].

From a social justice perspective, our results suggest no systematic biases in access to emergency medical services. After adjustment for marital status (household structure) and geographic location, there were no strong effects of social class, immigrant status, or age on risk of dying prior to transport vs. during or after transport. We interpret the protective effect of minority race/ethnicity, compared with being white, on unmeasured differences in household structure that were not captured by marital status. Specifically, racial/ethnic minority adults, particularly Hispanics and Asians, are more likely to live with family members compared with whites in the United States [26-28]. In addition, there may be cultural and geographic variations in attitudes, practices, and acceptance of death outside of hospital that could explain the regional and racial/ethnic findings in our study.

In interpreting our findings, it is important to remember that we did not measure heart disease mortality rates in this study. Consequently, for example, the observed result that African American cardiac decedents were less likely to die with no transport than similar white cardiac decedents does not mean that the overall cardiac death rate is lower for African Americans than for whites. Persistent racial and ethnic disparities in heart disease mortality are well established in the public health literature [29,30]. The same is true for social class. In our study, decedents of lower social class were slightly less likely to die with no transport compared with decedents with at least a high school diploma. Yet heart disease death rates have been shown in both U.S. and international studies to be higher among persons of lower social class [11,31-33].

However, in the case of marital status, our findings *do *suggest a testable hypothesis to explain previous research findings which consistently show higher heart disease death rates for single, divorced, and widowed persons compared with married persons [34-38]. Higher cardiac death rates for unmarried vs. married persons could be due, in theory, to higher incidence of heart disease[39,40] or to higher case fatality [37], or both. Our study suggests that at least some portion of this excess is due to higher case fatality: unmarried persons who live alone may have impaired access to life-saving emergency medical services. A future study which examined the unmarried excess in cardiac death rates for no transport vs. post-transport could provide evidence to support or refute this hypothesis.

One recent publication [19] reported results of a study very similar to ours. Data from the National Longitudinal Mortality Study (NLMS) was used to compare characteristics of cardiac decedents who died in-hospital vs. out-of-hospital (n = 59,034). The investigators included deaths with place of death = *dead on arrival *as out-of-hospital, whereas in our study these deaths were included with in-patient deaths under the category "post-transport." It should also be noted that in the study by Sorlie et al, deaths occurred during 1979–1989, whereas our study examined deaths that occurred in 1999–2000. Differences in results between these two studies could be due to methodological differences (e.g. sample data vs. national vital statistics data) or to secular trends in the relative importance of specific sociodemographic characteristics. In contrast to our study, these investigators found an excess risk of out-of-hospital (i.e. no transport) cardiac death for non-metropolitan residents and African Americans, and no excess risk in the Western region. However, many results were similar to ours, particularly the findings of no effect of social class and a statistically significant excess risk for unmarried vs. married decedents. Furthermore, the NLMS dataset provided a direct measure of household size, and the study found an excess risk of dying out-of-hospital for persons living alone, after controlling for age, sex, race/ethnicity, region, rurality, education, income, and marital status.

### Limitations and directions for future research

There are a number of challenges in designing studies to understand and prevent the phenomenon of cardiac death prior to transport. The first and most obvious challenge is that these deaths occur for the most part without medical or professional witness. Consequently symptoms and signs prior to death are unknown, or known only to lay witnesses[24]. The cardiac victim's thoughts, beliefs, and actions in reaction to symptoms and signs are mostly unknown. The presumption that the clinical course of cardiac events in people who die before transport is fundamentally the same as in people who survive to hospital admission or discharge may be a plausible presumption, but until innovative research provides an empirical view of these fatal cardiac event histories, it must be recognized as only a presumption.

Previous research has typically focused on one aspect or dimension of the entire cardiac event history picture. The most convenient type of study to perform is a study of heart attach survivors, either hospital or community-based. These studies have provided valuable insight into cardiac patient experiences and perceptions of symptoms and signs, use of emergency services, and barriers to medical care [14,15]. However, survivor studies by design exclude the highest risk and most vulnerable cases from their study populations, i.e. persons who do not survive long enough to be admitted to hospital. Our current study, presented in this paper, provides valuable information about the characteristics of these decedents but consequently lacks information about short-term survivors of major cardiac events. Furthermore, vital statistics data are limited due to the potential for misclassification of cause of death. Misclassification is greatest for specific causes (e.g. ''sudden cardiac arrest''); using a broad definition of heart disease as we chose to do minimizes misclassification but also compromises specificity.

The ideal study design would focus on a geographically-defined community and would, through multiple linked sources of surveillance, identify all acute cardiac events that occur in the community. An "event history" would be ascertained for each person, including the following elements: (1) date, time and location of the initial event; (2) eyewitness accounts from the victim (if a survivor) and any lay witnesses; (3) a record of lay witness resuscitation attempts prior to EMS arrival; (4) a complete record of 911 calls, EMS/ambulance response and treatment; (5) a transport history for the victim, including timing, source of transport (self, lay witness, EMS); (6) complete hospital records, including emergency department and in-patient records; (7) death certificates for victims who do not survive; (8) autopsy data when available; (9) short-term follow-up for victims who survive to hospital discharge; (10) linking of event histories by person to capture multiple acute cardiac events suffered by a person during a defined time period. The challenge, of course, is that by its nature the ideal study would be quite expensive to implement and would require cooperation and coordination among multiple organizations (primary care providers, EMS, health departments, and hospitals). However, the ideal study, if implemented correctly, would provide critically important data to identify persons with heart disease most vulnerable to dying prior to transport.

## Conclusion

Our results suggest that marital status, a broad marker of household structure, social networks, and social support, is more important than social class or race/ethnicity as a predictor of access to emergency medical services for persons who suffer an acute cardiac event. Regionally, we found an excess risk of dying prior to transport among cardiac decedents in the western United States. Future research should focus on ascertaining "event histories" for all acute cardiac events that occur in a community, with the goal of identifying the most important predictors of which individuals and neighborhoods are most susceptible to cardiac fatalities prior to medical intervention and transport.

## Abbreviations

AED = Automatic external defibrillator

CPR = Cardiopulmonary resuscitation

EMS = Emergency medical services

ICD = International Classification of Disease

MI = Myocardial infarction

NLMS = National Longitudinal Mortality Study

OR = Odds ratio

SCA = Sudden cardiac arrest

## Competing interests

The author(s) declare that they have no competing interests.

## Authors' contributions

All the authors collaborated on the conception and design of the study. EB and SR conducted the statistical analyses. EB drafted the manuscript. All authors read and approved the final manuscript.

## Pre-publication history

The pre-publication history for this paper can be accessed here:


